# Greenhouse Gas Emission Reduction Potential of European Union’s Circularity Related Targets for Plastics

**DOI:** 10.1007/s43615-022-00192-8

**Published:** 2022-07-14

**Authors:** Anna Tenhunen-Lunkka, Tom Rommens, Ive Vanderreydt, Lars Mortensen

**Affiliations:** 1 VTT Technical Research Centre of Finland Ltd, Tekniikantie 21, 02044 Espoo, Finland; 2 VITO, Boeretang 200, 2400 Mol, Belgium; 3 EEA European Environmental Agency, Kongens Nytorv 6, 1050 Copenhagen K, Denmark

**Keywords:** Plastics, Plastic value chain, Circular economy, Climate change, Greenhouse gas emissions, Climate change mitigation, Carbon footprint

## Abstract

Current rising concerns about environmental and climate impacts in production, consumption and end-of-life of plastics have led to efforts to switch from linear to circular economy of plastics in Europe. Greenhouse gas emissions are likely to decrease with a transition to a circular system; however, a systematic and integrated perspective on plastics and the carbon cycle is currently missing in the debate on plastics.

In this study, a model to estimate greenhouse gas emissions of the current mostly linear plastics value chain of the EU in 2018 and a future scenario, 2025 model, were created. By 2025 if current policy targets are reached, the plastic packaging recycling rate should be 50%, PET-based drinking bottles should include 25% recycled content, 77% collection target for plastic bottles, 10 Mt recyclates should enter the markets, uptake of bio-based plastics is estimated by European bioplastics to increase from current 1 to 1.32% and landfilling will continue to decrease according to the current trend at 3.85%.

Total greenhouse gas emissions caused by the current plastics value chain are estimated at 208 million tonnes of CO2-eq. The 2025 model estimates that total plastics value chain emissions will be 182 Mt of CO2-eq. Reduction potential is approximately 26 Mt of CO2-eq or 13%.

## Introduction

Plastics are one of the world’s most used materials in a wide range of different applications like packaging, electronics, automotive and construction. In 2017, 348 million tonnes of plastic were produced worldwide. The consumption of plastics is expected to rise globally and estimated to quadruple by 2050 [1, 2]. In Europe, 25.8 million tonnes of plastic waste are generated annually, of which less than 30% is collected for recycling and significant shares are exported from EU to be treated elsewhere. Landfilling (31%) and incineration (39%) of plastic waste are predominant treatment methods, and by these treatment options the valuable materials are lost from circulation. In the EU, it is estimated that 95% of the value of plastic packaging material, between 70 and 105 billion EUR, is lost annually after a very short first-use cycle. Globally, it is common that plastic waste is not well managed nor kept in circulation. Of all the plastics made between 1950 and 2015, it is estimated that only approximately 6% has been recycled and 1.2% of those recycled plastics are still in use. So, nearly 80% of discarded plastics are left to accumulate in landfills or the natural environment [3]. The inadequate management of plastic waste has thus resulted in plastic waste leaking into the environment causing environmental, societal and economic implications.

Rising concerns about the sustainability of current plastics production, consumption and end-of-life practices have led to global efforts to find ways to mitigate the environmental impact of plastic products, more in particular those related to littering and greenhouse gas (GHG) emissions. More and more initiatives are developed to support the switch from a linear to a circular economy for plastics. Especially in the European Union (EU), different strategies and targets have been set to initiate and reach the circular transition. A circular economy should indeed lead to plastic waste reduction and more efficient resources use. But an important underlying assumption is also that it would reduce greenhouse gas emissions in the value chain, and in this respect contribute to the climate targets. The linear plastics value chain is estimated to have significant GHG emissions, which contribute to climate change. GHGs are known to be released throughout the lifecycle of plastics: from the extraction of resources, through the refining and processing of the feedstock into resins, the conversion of plastics into products and components, during the products’ use phase and eventually on their end-of-life pathway, which for example ends with incineration. However, the overall level of GHG emissions from the plastics value chains is unclear. In 2019, the carbon footprint of global plastic production and incineration—not the whole value chain—was estimated at 850 Mt of CO_2_ [4].

Considering the foreseen global growth in plastic production and use, these emissions of 2019 at 0.85 gigatonnes (Gt) carbon dioxide equivalent (CO_2_-eq) could reach to 1.34 Gt by 2030 and 2.80 Gt by 2050. Based on the production volumes, the European Union’s contribution to this global impact could be 15 to 20% [4]. In 2018 the total annual global emissions of GHG were estimated by the International Panel on Climate Change (IPCC) to be 55.3 Gt CO_2_-eq [5], of which plastics contributed roughly about 1.5%. This was calculated by comparing the CIEL’s 2019 figure to the IPCC’s 2019 global total figure. The overall GHG emissions are expected to decrease when comparing the current mostly linear system to a more circular future system. However, a systematic and integrated perspective on plastics and the carbon cycle is missing. It is complicated to analyse the plastics value chain on a material and product level as the flows and chains are intricate and the allocation of emissions is challenging. Nonetheless, from a scientific perspective it is interesting to look at a holistic input–output approach to identify pain points or bottlenecks where attainable and detectable improvements or results could be obtained.

The European Commission (2019–2024) plans to become carbon neutral by 2050 and make the EU a leader on reducing the use of single-use plastics. In the European Strategy for Plastics in a Circular Economy [6], the European Commission highlighted that plastics are an important everyday material produced and used in EU’s economy and its citizens’ daily lives, but also emphasises the urgency to solve the environmental issues. Furthermore, the EU 2020 New Circular Economy Action Plan emphasises actions to increase circularity and reduce the environmental impacts of plastic, and the EU Single-use Plastics Directive provides specific legislation to solve single use plastic-related overconsumption. Given the rate of fast-growing global plastic demand, it is expected that the plastics industry will continue to use virgin fossil-resources to provide a large share of the demand. However, recyclates are perceived as key enablers for carbon–neutral solutions for the plastics value chain. Hence, the conversion of plastic waste into recyclates and new products is widely supported in EU policy documents and industry-led initiatives.

The European Green Deal sets ambitious objectives to the European plastics value chain by setting reduction goals for GHG emissions, both in the short and long term, and defining that all packaging needs to be reusable, recyclable or compostable by 2030 [6, 7]. The European plastics strategy concludes that in the EU the potential for recycling plastic waste is unexploited, and reuse and recycling activities are very low when compared to other materials like metals. According to the European Court of Auditors, there is a significant risk that EU will miss its plastic recycling targets if the efforts are not ramped up. Finding a way to make plastics compatible with a low-carbon economy is an acute challenge, and various opportunities have been identified to reduce the carbon intensity of the plastics system.

## Goal and Scope

This manuscript presents an extension to the work of ‘*Greenhouse gases and natural capital implications of plastics and bioplastics*’ [8] of the 2020 ETC/WMGE work programme by the same authors. To reinforce the understanding of the links between the circularity of plastics, the European plastic value chain, from the extraction of raw materials through production and use to the end-of-life waste treatment of plastics, was analysed from a lifecycle perspective. The focus of this analysis is the impact of all steps in the total plastics value chain across Europe, including feedstock production, refining, cracking, compounding, manufacturing and waste management. Because of the diversity of plastics and the number of applications in which they are used, the impacts of the use phase were not included. It was assumed that for a vast majority of plastic applications, emissions during the use phase can potentially be neglected, because they are most likely trivial or insignificant compared to the other life cycle phases.

The basic 2018 GHG emission scenario model and inventories for GHG factors have been produced within the aforementioned ETC/WMGE work programme. The extension presented in the current manuscript consists of the future 2025 model and the estimation of the GHG emission reduction potential of the EU’s Circular Economy targets. The overall goal of the study was to map plastic flows and related CO_2_ emissions for different parts of the total plastics value chain in the EU. The aim was also to better understand GHG emissions related to the plastics value chain as it transforms from a largely linear system to a more circular system. Current (mostly linear) and future (more circular) models were established to provide the insights needed to inform future discussions on the potential and limitations of circular plastics and the corresponding impacts on climate.

The model to estimate GHG emissions caused by the current—mostly linear—plastics value chain of the EU was based on data for 2018. A future scenario was built to estimate the overall GHG emissions in 2025, assuming that the plastics value chain should have changed according to the commitments and targets laid down in the European Plastics Strategy and European regulation. These targets are described in the European Plastics Strategy, or put forward by the Circular Plastics Alliance, Plastics Europe, nova Institut and European Bioplastics as follows:

- 10 Mt of plastic recyclates should enter the markets [6, 9];
- recycling rate of plastic packaging should be at 50% [10];
- there is a 77% collection target for plastic bottles [11];
- plastic drinking bottles should include 25% recycled content [11];
- the market uptake of bio-based plastics increases up to 32%, from a current share of 1% of all plastics [12, 13];
- landfilling will continue to decrease on the trajectory of 3.85% for the next 5 years [14].

## Materials and Methods

### Overview of the Methodology

Very few attempts have yet been made to map total material flows and related CO_2_ emissions for the total plastics value chain as it is very complicated to track the fate of carbon-containing resources through feedstock and polymer production to a variety of plastic products in use with different lifetimes and end-of-life options. In this study, two models were established to assess the GHG emissions related to the current plastics value chain and the future scenario in the EU. The current situation of the EU’s plastics value chain is based on figures for the 2018 model, and a future scenario model, the 2025 model, is built based on targets and trends associated to the transition towards a circular plastics economy in the EU.

The methodology for GHG factors and the current 2018 model was developed within the task on ‘*Greenhouse gases and natural capital implications of plastics and bioplastics*’ of the 2020 ETC/WMGE work programme [8]. GHG emissions caused by the European plastics value chain are estimated using a ‘bottom-up’ approach. It starts from plastic production, consumption and waste management data. For each lifecycle step, GHG emission factors are then attributed to the main flows and processes, which then allow to calculate the emissions throughout the value chain. The use phase was not analysed as part of the models as emissions and credits can be challenging to assign; the use phase-related emissions are also estimated generally to be quite low when compared to the big picture [8]. 

GHG emissions are expressed assuming a 100-year time horizon for the cumulated integration of the IR (infrared radiation) of the emitted GHGs, applying the IPCC 2013 methodology [15]. This means that not only CO_2_ emissions, but also other greenhouse gases—methane, nitrous oxide, etc.—are included. These other GHG vary in their relative contributions to global warming. The difference is expressed by the global warming potential (GWP), which is a factor used to calculate the contribution of other GHG in terms of CO_2_ equivalents [8]. 

The overall methodology to produce both of the scenario models is based on the following steps:

- An inventory of average GHG emissions (based on cradle-to-gate life cycle inventories) for fossil- and bio-based polymers was assembled based on a literature review and case studies [8].
- For polymer conversion processes, GHG factors were estimated based on LCA literature [8]. End-of-life options (landfilling, incineration, recycling, composting) were evaluated and matched with GHG factors, including avoided emissions for incineration and composting. The same end-of-life-management GHG emission factors were used for both scenarios (current and 2025) and for both fossil- and bio-based plastics [8].
- Parts of the value chain (production, conversion, and end-of-life) were matched with the EU’s respective mass flows (for different polymers, end-of-life management option, etc.) in each of the models. The 2018 model was based on actual data, and 2025 model based on the set targets and seen/expected trends. The volumes of bio-based plastics’ end-of-life management were based on the current situation that 55% is compostable and 45% is not. The 45% that is not composted is divided into landfilling, incineration and recycling as the fossil-based plastic waste flow today [8].
- To obtain total annual GHG emissions for the European plastics value chain, CO_2_-intensity factors were multiplied with mass flows obtained from sectoral data for polymer production and conversion, and waste processing.

The plastics value chain is both very diverse and globally spread. Oil and gas, which are still the principal feedstock for plastics production, are sourced in different parts of the world. Polymers are produced in the EU, not only for the EU market, but also for export, and imported [8]. 

The total primary production of polymers in the EU is estimated at 61.8 Mt per year. Polyethylene (PE) and polypropylene (PP) represent by far the largest volume, followed by polyvinylchloride (PVC), polyurethane (PUR), polyethylene terephthalate (PET) and (expanded) polystyrene ((E)PS) [8]. 

These polymers are converted into plastic products, which are sold in the EU, but again, also exported, and imported. Similarly, plastic products are sold on the EU market or exported to other markets or imported. Finally, plastic waste, collected for recycling, is partially exported for processing outside the EU [8]. The EU converters demand of around 51.2 Mt per year is smaller than polymer production, meaning that the EU is a net polymer exporter [1].

Finally, plastic waste, collected for recycling, is partially exported for processing outside the EU. There is, however, a large gap between the volume of plastic used in products each year, 51.2 Mt, and the yearly volume of plastic waste collected, 29.1 Mt [8]. There are several reasons for this imbalance: products have variable lifetimes, not all waste is collected properly, and not all plastic products produced in the EU are sold, used and discarded in the EU itself.

The current study has its limitations, both with respect to the approach and the scope. For reasons of clarity, these limitations are listed explicitly:

- The report does not claim to provide a complete or partial life cycle assessment *strictu *sensu, nor to follow any official assessment procedure or technical standard;
- The reported GHG emissions always refer to a combination of direct, and indirect and avoided emissions, regardless the place where these emissions occur, so they should be considered as global emissions, contributing to the environmental burden embedded in the plastics;
- Avoided emissions, both for output products from incineration and recycling, are explicitly excluded from the scope;
- Due to the lack of data, data accuracy and the various data sources used, detailed analyses of uncertainty and sensitivity are not possible to do. The data quality, its limitations as well as model uncertainties were analysed by conducting an overview of the uncertainty and sensitivity and are presented in chapters 4.2, 5.1 and 5.2.

### Current Model (2018)

The 2018 model was developed within the task on ‘*Greenhouse gases and natural capital implications of plastics and bioplastics*’ of the 2020 ETC/WMGE work programme [8]. In order to obtain a snapshot of the European plastics value chain and related GHGs, the scope for this analysis was defined as follows [8]:

- 2018 is the reference year.
- The total GHG emissions calculated for the EU plastics sector in this study are those related to the volumes converted on the EU market. The volume of polymers produced for the EU market is assumed to equal the EU converters demand: 51.2 Mt per year. If converters import some plastic polymers from outside the EU, the carbon footprint of these is assumed to be the same as the carbon footprint of polymers produced in the EU. GHG emissions related to energy use in the production and conversion steps are based on figures which reflect the average EU energy mix of fossil-based, nuclear and renewable energy [8].
- No avoided impacts are allocated to the use of secondary materials (recycled plastics) [8];
- PET-fibres, polyamide (PA)-fibres and polyacryl-fibres are not included in the study as they are not included in the statistics figures from Plastic Europe (2018). We did not include fibres in Europe because they are not in the statistics that came from Plastics Europe, and we did not find any other good public sources that would allow us to derive the volumes of polymers used for. The European production of man-made fibres is estimated by the European Man-Made Fibre Association at 3.5 Mt in 2018. Since the overall tonnage is estimated to be rather small compared to the total volume of polymers, the fibre applications were excluded from our analysis [16].
- The potential impact of plastic products made outside the EU but sold, used or discarded in the EU was not taken into account. In this simplified approach, the plastic conversion of 51.2 Mt per year in the EU was taken as plastic use. No GHG emissions were allocated to the use phase [8].
- The GHG emissions in the end-of-life phase were based on plastic waste collection figures, 29.1 Mt, which was applied with sorting and recycling residues and losses. The losses and residues (3.4 Mt) were incinerated. Plastics collected for recycling were assumed to be recycled in the EU via mechanical recycling. Additional impacts from long-distance waste transport, for example, were not reflected in the result [8].
- Avoided emissions were taken into consideration for energy production in electricity and heat production; 30 kJ/kg for lower heating value (LHV) of plastic waste was used. It was assumed that all incineration is with energy recovery based on the European overall waste-to-energy installations average energy efficiency 13.7% and heat efficiency 31.8% [8].
- Plastics Europe (2018) statistics for Recycling (9.4 Mt), Incineration (12.4 Mt) and Landfilling (7.2 Mt) were used [1].

### Future Model (2025)

For this study, model for 2025 is based on reaching the set targets. To investigate the model uncertainty, pessimistic and optimistic scenarios were created as well.

The following hypotheses were assumed:

- Plastics Europe has estimated that the primary production has decreased 0.5% from 2018 to 2019 [14]. In the beginning of 2020, a drop in primary production of plastic in EU was seen due to COVID-19. The production levels are expected to increase post-COVID-19 [17]. However, changes due to the SUP directive are expected to decrease the overall plastic consumption, as well as different reuse strategies. But on the other hand, it is expected that the consumption of plastics will increase with the growing gross domestic product (GDP). The GDP growth is estimated to be 1.65% in 2025. Due to these reasons, it was assumed for the 2025 model that the plastic demand would remain the same from 2018 figures at 51.80 Mt. Feedstock for production and conversion is based on fossil virgin plastics (41.10 Mt), recycled fossils plastics (10 Mt) and bio-based plastics (0.70 Mt). It is estimated that collected waste will remain on a similar level at 29.10 Mt, but most likely it will increase in the future. The plastic demand, plastic waste collected and the end-of-life fate volumes were modified for the pessimistic and optimistic scenarios.
- To achieve the 10.00 Mt recycled polymers target, 13.00 Mt of plastic waste should be collected and sent to recycling taken into consideration residues and losses from sorting and processing. On average, approximately 30% of plastic waste collected ends up as rejects and residues during collection, sorting and recycling. This would mean that 3.00 Mt of collection and recycling rejects and residues is sent to incineration.
- 10.00 Mt of recycled polymers was accounted for commonly recycled polymers and allocated to the following categories, in accordance to the targets of plastic packaging separate collection and recycling, and targets set for plastic bottles (PET) recycled content: low density (LD) PE, high density (HD) PE, PET, PP and others. The ‘others’ category includes fossil-based polymers other than PP, PS/EPS, LD and MD PE, PVC, PET, PUR. Recycling GHG-emission factor is for mechanical recycling like in the 2018 model. Chemical recycling was not taken into consideration in the calculations.
- Recycling rate of plastic packaging is set at 50% for 2025 [10].
- Collection rate of 77% for plastic bottles in 2025 [11].
- By 2025, plastic drinking bottles should include 25% recycled content [11].
- Avoided emissions from virgin polymer production were taken into account by excluding production related emissions for 10.00 Mt of recycled polymers. The GHG-factor was 2.25 kg CO_2_-eq./kg polymer. Based on the estimates in literature, the avoided emissions from virgin polymer production from fossil resources range from 1.91 to 5.70 kg CO_2_-eq./kg polymer, and recycling rather than incinerating plastics could reduce emissions by 1.10–3.00 tonnes CO_2_-eq./tonne plastic compared to plastics from virgin fossil feedstock [18].
- The EU has set as a target that recyclable materials in general should not be landfilled. The Landfill Directive will prohibit sending more than 10% of total mixed solid waste to landfill after 2035. Due to the fact that there is no specific target for 2025, a trajectory of − 3.85% decrease in landfilling plastic waste is adopted to the model based on Plastics Europe 2019 data source. This means that landfilling is estimated to further decrease by 3.85% by 2025, which would equal to an additional 6.90 Mt sent to recycling. For comparison, landfilling of municipal waste is set to decrease to 10% by 2035 [19].
- The rest of the collected waste, 8.96 Mt, is sent to incineration. By combining the residues and rejected plastic waste (3 Mt), the total portion of incinerated plastic waste would be at 11.96 Mt. This would mean that the amount of plastic waste incinerated in 2025 would be less than in 2018.
- Globally, 1% of all plastics are bio-based. A market estimation by nova Institut and European Bioplastics predicts that the range of bio-based plastics in Europe will increase by 32% [12, 13]. Thus, for the 2025 model, it was estimated that the bio-based plastics share would be 700 kilotonnes. The biggest Compound Annual Growth Rate (CAGR) was expected for PLA, PBAT and bio-PE, so those were used in the model. Currently, about 55% of bio-based plastics on the market are biodegradable; thus, an estimate was made that 45% of the bio-based plastics of the collected bio-based plastics would go through the same processes as conventional plastics at that time—recycling, incineration and landfilling—and that the remaining 55% would be composted. The scenario estimation for composting is done on the current situation of what would be possible to compost; this does not reflect on the future trends of end-of-life-management of bio-based plastics. The avoided emissions for composting were estimated at − 0.133 kg CO_2_/kg waste [20].
- Additional scenarios for 2025 were created to estimate the uncertainty of the model and make assumptions. The 2025 model is developed so that the targets would be met as set. A pessimistic scenario is based on that the set targets would not be met resulting in less recycling, more incineration and landfilling, plastics production amount increase slightly, plastic waste ending up in end-of-life-management increase very moderately. The optimistic scenario is based on that the set targets would be exceeded, which would result in higher amount of recycling and available recyclates in the markets, virgin plastic production overall volumes would be less, landfilling and incineration decrease and the share of plastic waste ending up in end-of-life-management increases slightly more. The varied parameters against the 2025 model are as presented below and have been set based on reflecting on the current status and its trajectories and authors’ pragmatic estimations on the optimistic and pessimistic situations.

### Production of GHG Factors for the Models

Plastics value chains were evaluated from the perspectives of the following life cycle stages [8]:

- Production phase: resource extraction, refining and polymer production, compounding.
- Conversion: manufacturing of components and products from polymers.
- End-of-life management: recycling, incineration, landfilling and composting.

It is clear that existing GHG emission datasets do not provide a complete picture of emissions at the level of product or material value chains. Therefore, a bottom-up approach was used. The basic data for this exercise are GHG emissions intensity factors that are found in lifecycle analyses and databases such as Ecoinvent. They express the level of emissions per (mass) unit produced, used, transported or treated (kg CO_2_-eq. per kg of material), including direct and indirect emissions per lifecycle phase (Table 1).

**Table 1:** Parameters varied for the pessimistic and optimistic scenarios (own assumptions)

Varied parameters	Pessimistic scenario	Optimistic scenario
Recycling Recycling residues, recyclates	− 10%	+ 20%
	*Recycling residues incinerated change according to the amount of recycled*	
	*Recyclates changed according to the amount of recycling*	
Plastics production amount	+ 5%	− 5%
Landfilling	+ 10%	− 10%
Incineration	+ 20%	− 10%
Plastic waste ending up in EOL	+ 2.5%	+ 5%

For the production and conversion phases, specific estimates can be made per polymer type. The primary focus was on high volume plastics, so common polymer types were distinguished: polypropylene, high- and low-density polyethylene, polyvinylchloride, polyurethane, polyethylene terephthalate and polystyrene. These polymers represent the vast majority of EU converters volumes. For other polymers, the median value of the others combined was used as an estimate (Table 2) [8].

**Table 2:** Greenhouse gas emission factors for polymer types, plastic polymer production and upstream processes (kg CO_2_-eq./kg polymer). Redrawn from source [16]

	GHG emission factor (kg CO_2_-eq./kg polymer) per life cycle phase			
Polymer type	Crude oil production	Refinery	Polymer production	Total production (cradle to gate)
PP	0.228	0.34	1.342	1.91
PE-LD	0.228	0.34	1.412	1.98
PE-HD	0.228	0.34	1.362	1.93
PVC	0.228	0.34	1.942	2.51
PUR (1)	0.228	0.34	5.132	5.70
PET	0.228	0.34	2.372	2.94
PS/EPS (2)	0.228	0.34	3.112	3.68
Other	0.228	0.34	1.942	2.51

For the end-of-life phase, no distinction was made between the fate of different polymer types. The main routes considered for plastic waste (fossil- and bio-based) are recycling, incineration, landfilling and composting. Avoided emissions are taken into consideration for incineration, − 0.976 kg CO_2_-eq./kg of material, and composting, − 0.1333 kg of CO_2_-eq./kg of material. For recycling, it was estimated that losses and residues from sorting and recycling are 20% at each stage (excluding collection and transport). For the remaining material, which was effectively recycled into secondary resources, a CO_2_ factor of 0.321 kg CO_2_-eq./kg was applied. Lost recycling residues were assumed to be incinerated (with energy recovery) and added to the volumes collected for incineration [8]. 

### a) Production Phase: Resource Extraction, Refining and Polymer Production

The production phase includes primary resource extraction (mainly fossil resources), refining of primary resources and polymer production. First, there are emissions related to crude oil production. Masnadi et al. (2018) estimated the emissions from 8966 oil fields in 90 countries, representing 98% of 2015 global crude oil and condensate production [21]. From this dataset, the global volume weighted average upstream carbon intensity (CI) was calculated to be 10.3 g CO_2_-eq./megajoule (MJ) crude oil. Using a heat value for crude oil of 42–47 MJ/kg, the global weighted value for crude oil production would be in the range of 0.432–0.484 kg CO_2_/kg crude oil [22].

When zooming in to the European plastics value chain, it is important to take account of the variable sources of crude oil and natural gas that are supplied to the European market. These supplies come from many countries all over the world, with different production practices, resulting in a wide range of carbon intensities: from 0.03 to 0.458 kg CO_2_/kg crude oil. The average carbon intensity for the EU cracker capacity mix is estimated at 0.228 kg CO_2_ emissions per kilogram of crude oil [22]. This is lower than the weighted global value, as calculated by Masnadi et al. (2018) [21]. Similarly, also the upstream natural gas supply chain has branches worldwide. For the EU cracker capacity mix, the average carbon intensity of the gas is estimated at 0.173 kg CO_2_ emissions per kilogram of feedstock [8]. 

The above data refers to the extraction of crude oil, which is used as a fossil resource for the production of plastics, but also for a variety of other chemicals and products. From a mass flow perspective, it is often complex to relate or allocate impacts in these up-stream processes to the down-stream flows, like—in this case—plastics. In the references used, such as the Ecoprofiles from Plastics Europe, this allocation has already been done.

As with crude oil production, the carbon intensities of global refining vary. Operating characteristics of different refineries depend on the type of crude oil they process and the demand for different fractions—naphtha, gasoline, etc. Liang et al. (2020) calculated the global volume weighted average of refining: 40.7 kg CO_2_-eq per barrel or 7.3 g CO_2_-eq./MJ [23]. Using a heat value for crude oil of 42–47 MJ/kg [22], the volume-weighted value for oil refining would be in the range of 0.307 to 0.343 g CO_2_-eq./kg crude oil. It is estimated that refining activities account for 40% of the emissions from the oil and gas supply chain, and 6% of all industrial emissions [23]. On average, more than 95% of the global refining carbon intensity is generated due to CO_2_ emissions, and only 4% due to methane [8]. 

Plastics Europe used data from seven oil refineries and world data from the International Energy Agency (IEA) statistics to compile the European eco-profile for naphtha [24]. To produce 1 kg of naphtha, it was estimated that approximately 1.1 kg of crude oil is used and according GHG emissions were estimated at 0.340 kg CO_2_-eq./kg naphtha, which is in line with the global figure from Liang et al. (2020). For each location of refineries and crackers, the respective country specific electricity mix, including the respective pre-chains, was used instead of an average EU electricity mix [8]. 

After refining, petroleum products are ready to be used as a feedstock for polymer production. The first step in the process is cracking the complex organic molecules in petroleum products into simpler ones. Several methods exist, of which steam cracking is one of the most common and widely spread. For this report, the scope was limited to the refinery itself, including all major process units, energy and hydrogen supplied to the refinery as well as the upstream emissions related to natural gas and electricity consumed by the refinery. In the EU, the main feedstock for crackers is naphtha: together with condensates from natural gas production, the share of naphtha in European cracker feedstock is estimated at 74% [25]. Other inputs are propane, butane and liquefied petroleum gas, 12%; gas oil, 6%; and ethane (refinery gases), 4%.

GHG emissions and other life cycle impacts related to polymer production are documented in Plastic Europe’s Ecoprofiles, which were published from 2005 to 2017 [25]. The same data can be found in the Ecoinvent database, for which the last update, version 3.6, was released in September 2019 [26]. These datasets provide figures for the production of a range of polymers, including aggregated data for upstream processes. Impacts related to electricity and/or heat use are tailored to the EU market by using the European energy mixes [8]. 

The accumulated GHG emissions for the resource extraction, refining, cracking and polymer production stages range from 1.91 to 5.70 kg CO_2_-eq./kg polymer respectively for polypropylene and polyurethane rigid foam (Table 2) [8]. 

For bio-based plastics, there are several ways of refining and processing biomass, and in many cases, bio-based monomers can be produced in multiple ways as well. Typically, the raw material is refined into precursors, such as acids, glycerol or glucose, for the monomer production phase. These monomers are then polymerised and finally converted into plastic products.

A literature-based review of cradle-to-gate analyses of different biopolymers was carried out. Cradle-to-gate covers the raw material acquisition and pre-processing—the extraction of natural resources and the steps up to the components entering to the product production facility. The results vary considerably depending on the methodology used for calculations, whether they originate from specific case studies or were generalised/rounded for other purposes such as a general overview [8]. 

The literature review concluded that the GHG emissions of bio-based plastics are in the range of − 4.9–9.5 kg CO_2_-eq./kg of material. Negative emission results can imply for example that the emissions of bioproducts were considered, or release of biogenic carbon at end-of-life was not accounted for. The review results indicate that the overall impact varies between polymers and that within some solutions, especially bio-PE, there is a wide discrepancy between minimum and maximum values. Table 3 showcases the GHG emissions and their ranges for different biopolymers from cradle to gate based on reviewed case studies [8].

**Table 3:** Greenhouse gas emissions for bio-polymer types (kg CO_2_-eq./kg polymer). Source: Based on data gathered from [27–36]

Polymer type	GHG emission factor (kg CO_2_-eq./kg polymer) production (cradle to gate): Average value [min;max]
Recycled PP	0
Recycled PE-LD	0
Recycled PE-HD	0
Recycled PET	0
Recycled other polymer	0
Bio PHA	0.12 [− 4.90;7.00]
Bio PBAT	3.11 [0.00; 9.50]
Bio PE	0.90 [− 2.50; 5.00]
Bio PLA	0.92 [− 1.30; 4.50]
Bio PP	− 0.06 [− 2.20; 1.86]
Bio PUR	2.75 [− 1.00; 7.00]
Bio PET	1.23 [0.00; 2.50]

### b) Conversion

The conversion step is about manufacturing components and products from polymers and basically involves the application of mechanical and heat energy. Lifecycle inventory data for conversion processes show that the additional GHG emissions caused by these processes are in the range of 0.294–1.14 kg CO_2_-eq./kg product for the extrusion of plastic pipes and stretch blow moulding, respectively (Table 4).

**Table 4:** Greenhouse gas emissions from plastic conversion technologies (EU averages) [8, 26]

Conversion technology and key contributing processes	GHG emission factor (kgCO_2_**-**eq**./**kg product)
Injection mould processing (Europe)	0.962
Blow mould processing (Europe)	0.917
Stretch blow moulding (Europe)	1.14
Calendering, rigid sheets (Europe)	0.322
Extrusion of plastic film (Europe)	0.416
Extrusion of plastic pipes (Europe)	0.294
Polymer foaming processing (RER)	0.513
Thermoforming with calendering (Europe)	0.642

Many of these conversion techniques can be applied to different polymers. Datasets which allocate certain conversion techniques to specific volumes of polymers in Europe are not available. Estimates for the share of conversion technologies used for the most common types of resin can however be found in the literature [37, 38]. Using these estimates, realistic assumptions were made regarding the application of conversion technologies for different polymers. GHG emission intensities were then calculated for the conversion of each polymer in the EU, by applying these shares on data from the Eco-invent 3.6 database (Table 5) [26].

**Table 5:** Estimated annual GHG emission factors from polymer conversion in the EU plastics value chain, 2018. Sources [8, 14, 37, 38]:

Polymer	Assumptions regarding conversion (adapted from Zheng and Suh, 2019)	GHG emission factors (kg CO_2_-eq./kg polymer)
PP	74% injection moulding; 24% blow moulding; 2% extrusion (pipes)	0.94
LD PE	67% injection moulding; 24% blow moulding; 9% extrusion (pipes)	1.13
HD PE	67% injection moulding; 24% blow moulding; 9% extrusion (pipes)	1.13
PVC	51% extrusion (pipes); 18% calendering (sheets); 29% injection moulding; 2% blow moulding	0.51
PUR	100% polymer foaming	0.51
PET	50% injection moulding; 50% blow moulding	0.94
PS**/**EPS	100% polymer foaming	0.51
Others		0.94

### c) End-of-life management

End-of-life management options for plastic waste are recycling, landfilling, incineration and composting.

Recycling is usually preceded by separate collection, sorting or pre-treatment, and several transport movements. Compared to the rest of the plastics value chain, the GHG emissions related to waste collection and transport are very small. The average CO_2_ emissions from the collection of waste by a specialised truck are about 0.0004 tonnes of CO_2_/tonne kilometre, so for a collection round of 40 km, the emissions per tonne of waste are 0.016 tonne CO_2_ [8]. Emissions from the transport following collection to the disposal site are about five times lower at 0.00008 tonnes CO_2_/tonne kilometre [15].

Table 6 gives an overview of the GHG emissions from plastic waste collection, sorting, transportation and recycling, as reported by Deloitte and Plastic Recyclers Europe [39] and Turner et al. [40]. The emissions from each step have to be added up to obtain a representative value for the recycling process itself. The recycling stage’s total emissions range from 0.414 to 0.576 tonnes of CO_2_-eq. per tonne of waste, depending on the polymer type and mainly related to the energy (heat, steam, electricity) needed for the recycling processes (Table 7) [8].

**Table 6:** Greenhouse gas emission factors from plastic waste collection, sorting, transportation and recycling. Source: [8], own calculation

Waste management step	Greenhouse gas emission factors (tonnes of CO_2_-eq./tonne of waste)	Remarks
Collection	0.017	Separate collection of plastic waste. Transport distances based on German averages (Plastics Recyclers Europe 2015)
Sorting/pre-treatment	0.027	Includes indirect emissions from dismantling and sorting of plastics from other recyclables in sorting facilities and may also include shredding and further sorting by plastic resin (Plastics Recyclers Europe 2015) Sorting leads to 20% residues, going to incineration
Transportation to recyclers	0.022	(Plastics Recyclers Europe 2015)
Mechanical recycling PET recycling HD PE recycling LD PE recycling PP recycling PS recycling PVC recycling Others recycling	0.510 0.348 0.348 0.348 0.348 0.348 0.348	Reprocessing sorted waste lead to 20% residues, going to incineration. Figures expressed as per ton output (Plastics Recyclers Europe 2015)
Total:	0.321 [0.269–0.373]	Average value [Min − max] values for (collection + sorting/pre-treatment + transportation + recycling), excluding treatment of residues, and per ton plastic waste input

**Table 7:** EU estimated annual greenhouse gas emissions from plastic waste management. Sources [8, 16]

EoL option		Greenhouse gas emission factors (kg CO2-eq./kg polymer)
Recycling		0.32
Incineration	Direct	2.73
	Recycling residues	2.73
Landfilling		0.03

These figures already take into account that both the sorting of the plastic waste and the reprocessing of the sorted waste lead to considerable amounts of residues, which have to be treated themselves once again and which are currently mainly incinerated. The share of residues of sorting and reprocessing plastic waste can be as high as 50% of the input material and typically amounts to on average between 20 and 50% [41].

During incineration the embedded carbon in plastic waste is completely oxidised to CO_2_. Starting from a carbon content of plastic waste of about 0.75 tonnes of C/tonne of waste [42], the corresponding European GHG emissions are estimated to be 2.70 tonnes of CO_2_-eq./tonne of plastic waste [43]. Depending on the specific polymer, this carbon content, and the corresponding emissions when incinerated, can differ slightly. Adding 0.017 tonnes of CO_2_-eq./tonne of waste for collection before incineration, the total emissions are estimated to be 2.71 tonnes of CO_2_-eq./tonne of waste [8]. As this emission factor only concerns the direct emissions from incineration, the indirect emissions from production of all necessary auxiliaries for the incineration, such as energy and flue gas cleaning additives, were added [8]. These indirect emissions are estimated at 0.05–0.10 tonnes of CO_2_-eq./tonne of plastic waste [44].

As the energy content of plastic waste is generally recovered during incineration (as electricity, heat or both), the incineration process is credited for the benefits from avoided production of conventional energy (electricity and heat) replaced by energy recovered from plastic waste incineration. These avoided emissions are calculated based on EU average energy efficiencies for waste incineration of 13.7% for electricity production and 31.8% for heat recovery, and on EU average data for conventional electricity and heat production (as in the EU GHG emission inventory and the Ecoinvent database). These credited or avoided emissions apply both to plastic waste sent directly to incineration and to sorting and reprocessing residues of plastic waste sent to recycling [8]. 

Recycling plastics decreases the demand of virgin feedstock materials. The benefit of recycling is therefore already included as a reduction of the actual demand of plastics, provided that the plastics are both recycled in Europe and the recyclate is used as a feedstock in Europe. Consequently, no avoided emissions are counted for in the case of recycling.

The EU has adopted a zero-landfill target to be achieved by 2030 for recyclable waste including plastics. The future options for plastic waste in the EU will therefore primarily be reuse, recycling or incineration. The available literature on GHG impacts of landfilling plastic waste gives a range of 0.004–0.010 tonnes of CO_2_-eq./tonne of plastic waste [39]. Assuming a value of 0.01 tonnes of CO_2_-eq./tonne of plastic waste and adding 0.017 tonnes of CO_2_-eq./tonne for the collection of the waste as explained, a value of 0.03 tonnes of CO_2_-eq./tonne for the landfilling plastic waste can be assumed [8]. The fate of plastics in landfills is not fully understood, and potential decomposition of plastics over hundreds of years might eventually lead to leakage of GHG emissions into the atmosphere. Legal or illegal fires on landfills can also lead to uncontrolled GHG emissions [8]. 

For biodegradable plastics, industrial composting can be an end-of-life option given that it is done the correct way. Composting results in CO_2_, methane and nitrous oxide emissions. Based on a study case, the GHG emissions from biodegradable polymers were on average 1.62 kg CO_2_-eq./kg of material [45]. This data was used. Yet, it is high when compared to recycling, incineration and landfilling emissions reviewed and used in this report [8]. Composting is sometimes also accounted for as carbon neutral due to sequestering during feedstock production. Existing research suggests that at least half of the compostable plastics is transformed into CO_2_ emissions in composting during biodegradation. For composting, the evidence is sparse and inconclusive whether the composting of plastics results in ecological improvements. Moreover, there seems to be a consensus on plastic composting resulting in a lack of nutritional benefits [45].

## Results

### Current Model (2018)

**Fig. 1:**
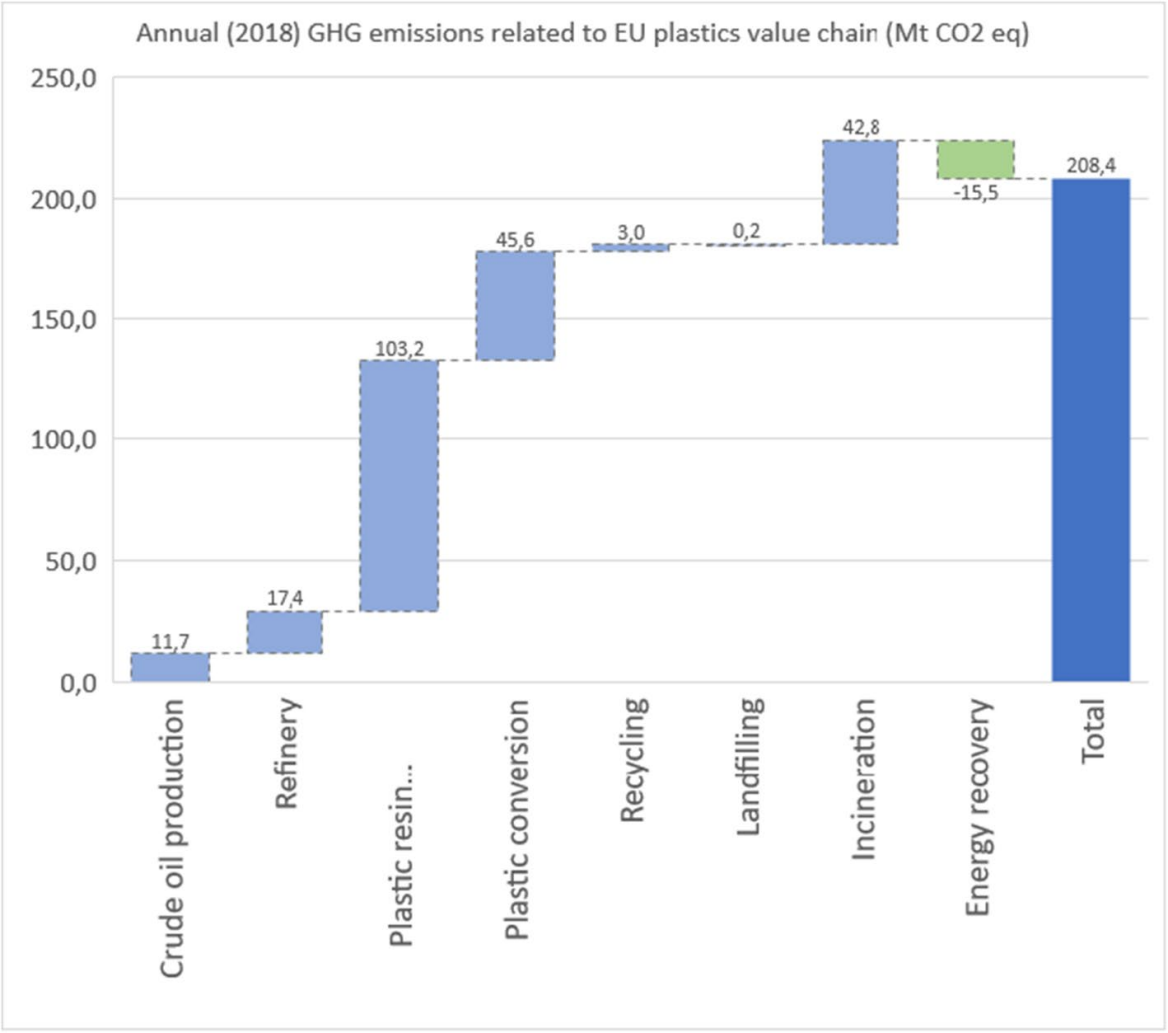
GHG emissions along the plastics value chain: 2018 model

**Fig. 2:**
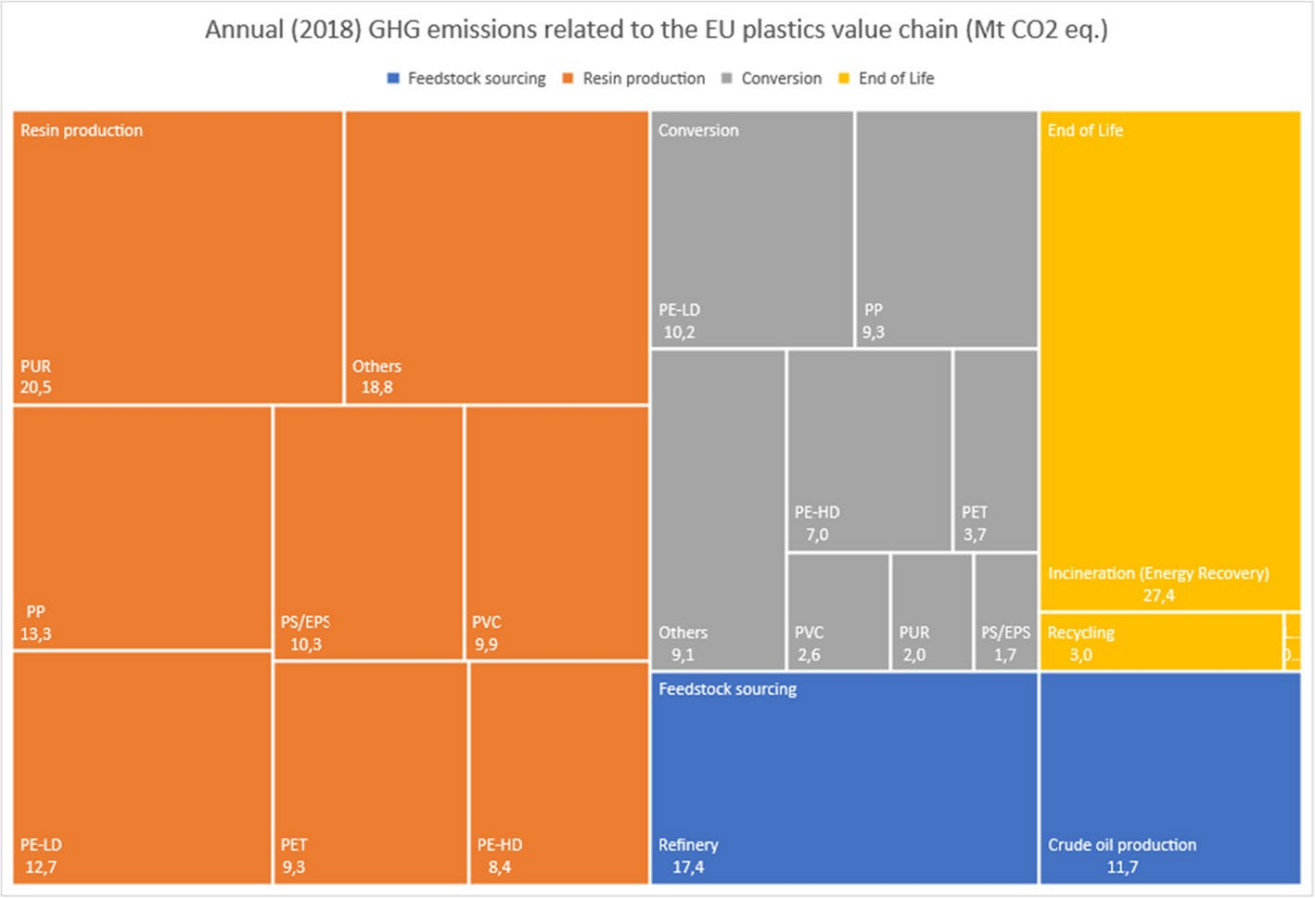
Annual (2018) GHG emissions related to the EU plastics value chain

The overall GHG emissions from the EU plastics value chain for 2018 were estimated at 208 Mt CO2-eq. (Table 8). The majority of this (63%) are embodied emissions, related to crude oil production and refinery, and resin production. Converting these polymers to products accounts for another 22% of the value chain emissions, and plastic waste treatment adds a further 15%, mainly due to incineration. The 2018 model is presented in Figs. 1 and 2.

**Table 8:** Estimated annual GHG emissions from the EU plastics value chain for 2018

Life cycle stage		EU converters demand (Mt)	Specific GHG emission factors (kgCO_2_**-**eq./kg polymer)	Annual GHG emissions** (**MtonCO_2_-eq)
Upstream resources	Crude oil production	51.1	0.228	11.65
	Refinery	51.1	0.34	17.37
Resin production	PP	9.9	1.342	13.29
	PE-LD	9	1.412	12.71
	PE-HD	6.2	1.362	8.44
	PVC	5.1	1.942	9.90
	PUR (1)	4	5.132	20.53
	PET	3.9	2.372	9.25
	PS/EPS (2)	3.3	3.112	10.27
	Others	9.7	1.942	18.84
Conversion	PP	9.9	0.94	9.31
	PE-LD	9	1.13	10.17
	PE-HD	6.2	1.13	7.01
	PVC	5.1	0.51	2.60
	PUR	4	0.51	2.04
	PET	3.9	0.94	3.67
	PS/EPS	3.3	0.51	1.68
	Others	9.7	0.94	9.12
EoL	Recycling	9.4	0.32	3.01
	Incineration (energy recovery)	15.8	1.73	27.36
	Landfilling	7.2	0.03	0.22
Total		51.1		208.43

Results show that the production phase for the polymers needed for further conversion in the EU causes emissions of approximately 132 Mt CO2-eq. More than 80% of these emissions are related to seven polymers: high- and low-density polyethylene, polyethylene terephthalate, polypropylene, expanded polystyrene, polyurethane and polyvinylchloride [8]. 

There is quite some variability in specific GHG emissions, depending on the polymer type and conversion technique. But overall, it can be concluded that, when a 1 kg plastic product comes onto the market, it has already caused at least on average 2.9 kg CO2-eq. emissions. Moreover, the same product will cause a further 2.7 kg CO2-eq. emissions when it is discarded and incinerated (not considering the avoided emissions for energy recuperation) [8]. 

### Future Model (2025) and Pessimistic and Optimistic Scenarios

**Fig. 3:**
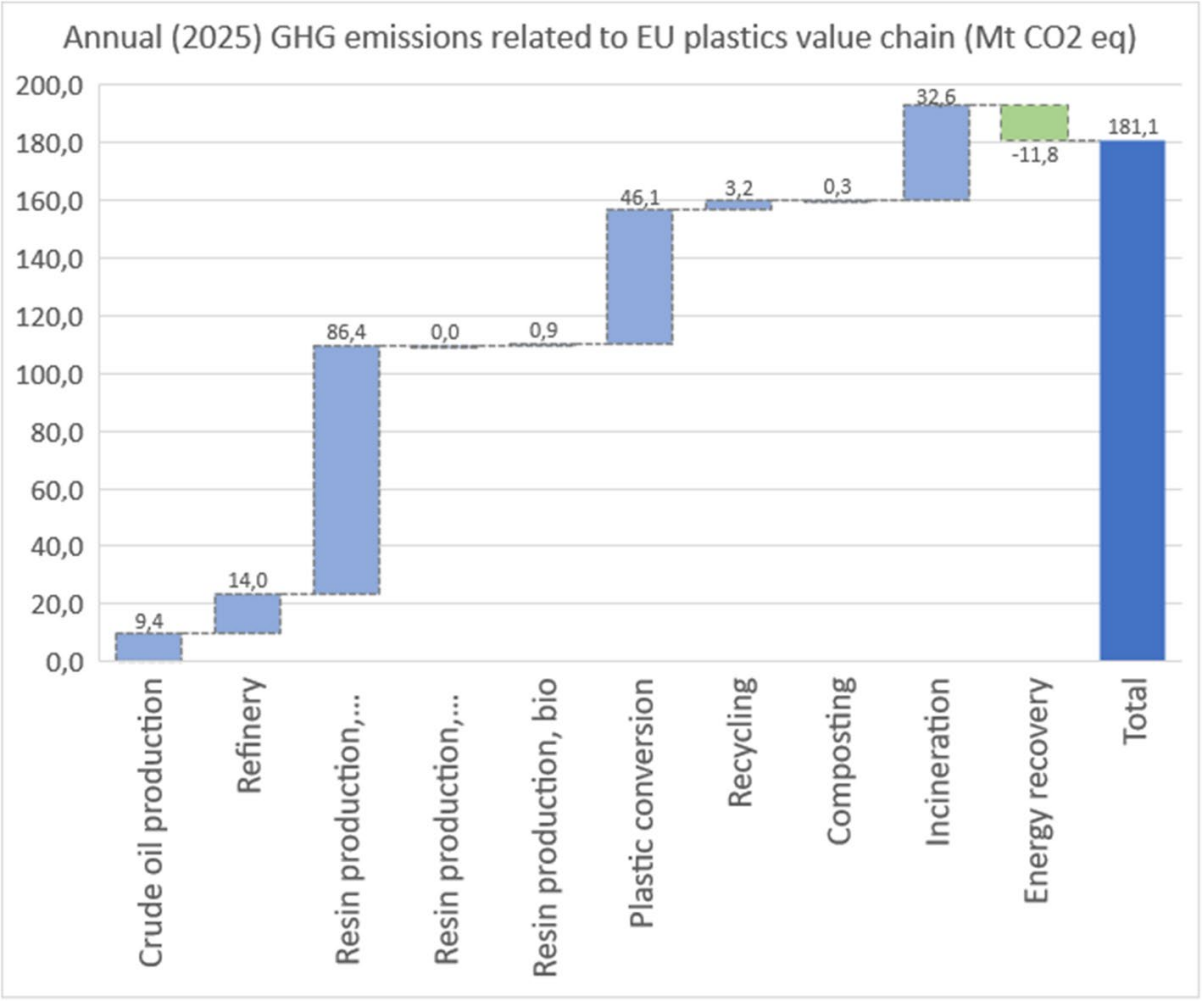
GHG emissions along the future plastics value chain: 2025 model

**Fig. 4:**
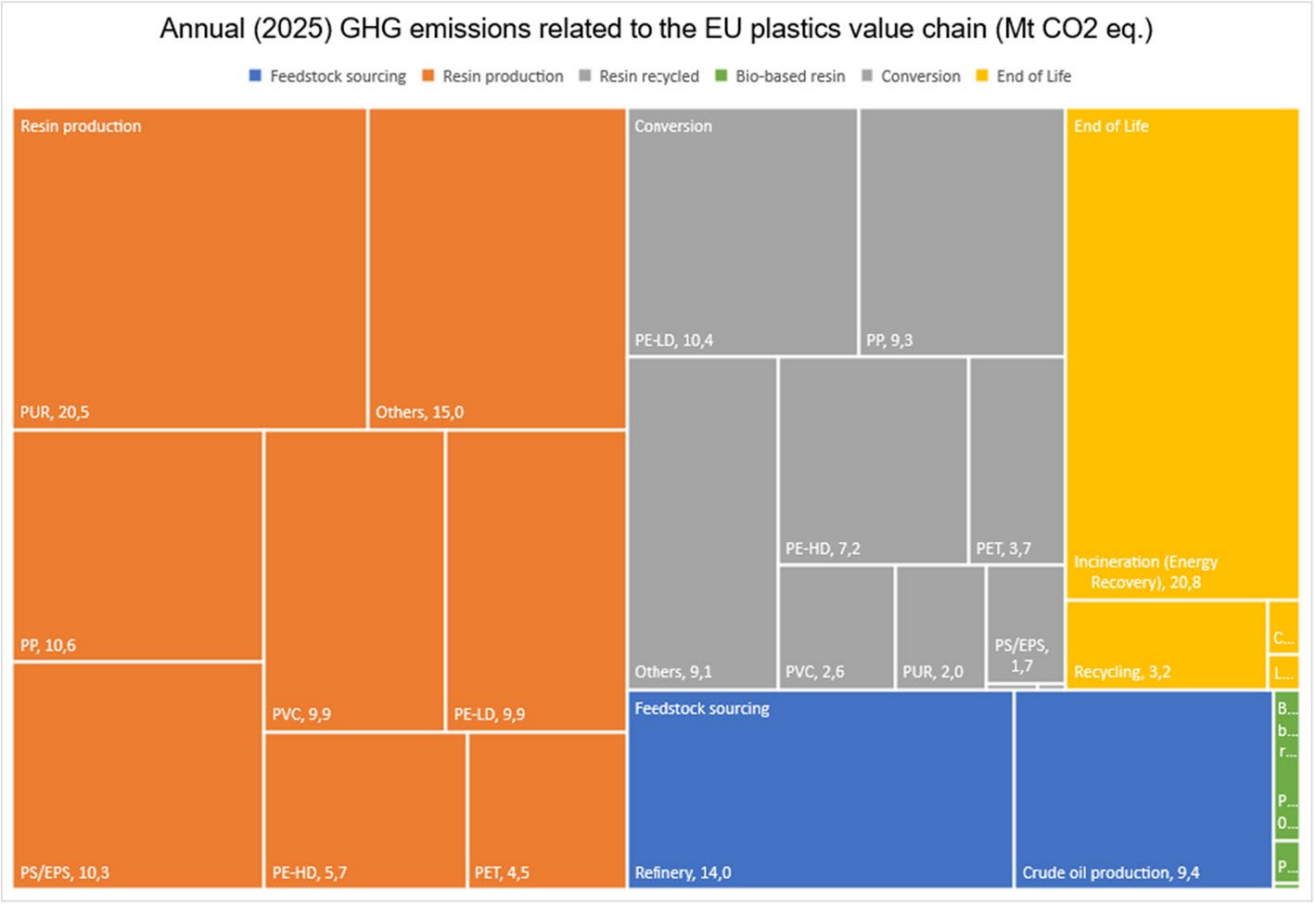
Annual (2025) GHG emissions related to the EU plastics value chain

The future 2025 model estimates that the total plastics value chain emissions could be 182 Mt of CO2-eq. The shares remain somewhat the same in the future model as in the 2018 model: 61% of GHG emissions are caused by production, 26% by conversion and 14% by end-of-life management. The data for 2025 model is presented in Table 9. The major savings in the future model comes from increased recycling and replacing fossil-based polymer production with recyclates. The future model is presented in Figs. 3 and 4.

**Table 9:** Estimated future annual GHG emissions from the EU plastics value chain for 2025

Lifecyclestage		EUconvertersdemand(Mt)	SpecificGHGemissionfactors(kgCO_2_**-**eq./kgpolymer)	AnnualGHGemissions(MtonCO_2_**-**eq)
Upstream resources	Crude oil production	41.10	0.228	9.37
	Refinery	41.10	0.34	13.97
Resin production	PP	7.90	1.342	10.60
	PE-LD	7.00	1.412	9.88
	PE-HD	4.20	1.362	5.72
	PVC	5.10	1.942	9.90
	PUR (1)	4.00	5.132	20.53
	PET	1.90	2.372	4.51
	PS/EPS (2)	3.30	3.112	10.27
	Others	7.70	1.942	14.95
Resin recycled	PP recycled	2.00	0	0.00
	LD PE recycled	2.00	0	0.00
	HD PE recycled	2.00	0	0.00
	PET recycled	2.00	0	0.00
	Other recycled	2.00	0	0.00
Bio-based resin	PLA	0.23	0.92	0.03
	PBAT	0.23	3.11	0.72
	Bio-PE	0.23	0.9	0.21
Conversion	PP	9.90	0.94	9.31
	PE-LD	9.00	1.13	10.17
	PE-HD	6.20	1.16	7.19
	PVC	5.10	0.51	2.60
	PUR	4.00	0.51	2.04
	PET	3.90	0.94	3.67
	PS/EPS	3.30	0.51	1.68
	PLA	0.23	0.51	0.03
	PBAT	0.23	0.22	0.05
	Bio-PE	0.23	1.13	0.26
	Others	9.70	0.94	9.12
EoL	Recycling	10	0.32	3.20
	Incineration (energy recovery) (incl. rec residue)	12.02	1.73	20.79
	Composting	0.22	1.49	0.33
	Landfilling	6.86	0.03	0.21
Total		51.8		181.31

The EU is targeting a 15% reduction in annual overall GHG emissions by 2025. This would mean that the 2025 total GHG emissions would be 3309 Mt CO_2_-eq. Of this, the plastics value chain would account for 5.5%. This is the same share as for the current 2018 model.

**Fig. 5:**
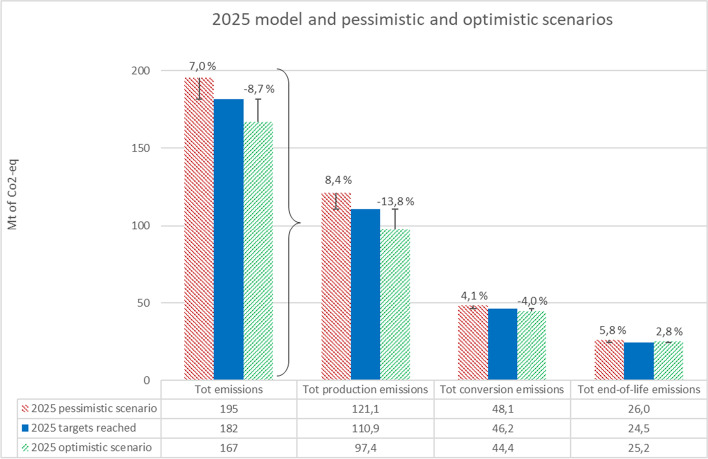
2025 model compared to the pessimistic and optimistic scenarios

The pessimistic and optimistic scenarios and their comparison to the 2025 model are presented in Table 10 and Fig. 5. For total emissions, the positive scenario resulted in further 8.7% decrease, and negative scenario in 7% less decrease when compared to the on target 2025 model. The biggest difference can be again seen on the total production emissions.

**Table 10:** Shares of total volumes and calculations used in the 2025 scenarios

		2025 pessimistic scenario	2025 on target model	2025 optimistic scenario
Production	Fossil virgin polymers (Mt of material)	44.69	41.10	36.51
	Recycled polymers (Mt of material)	9.00	10.00	12.00
	Bio-based polymers (Mt of material)	0.70	0.70	0.70
	Tot polymers (Mt of material)	54.39	51.80	49.21
	Tot GHG emissions (Mt CO_2_-eq.)	121.07	110.86	97.39
Conversion	Tot GHG emissions (Mt CO_2_-eq.)	48.14	46.19	44.43
End-of-life	Recycling (Mt of material)	9.00	10.00	12.00
	Recycling residues (Mt of material) (incinerated)	4.10	4.50	5.34
	Incineration (Mt of material of material)	8.96	7.47	6.72
	Composting (Mt of material)	0.22	0.22	0.22
	Landfilling (Mt of material)	7.55	8.68	6.18
	Tot EoL volumes (Mt of material)	29.82	29.10	30.45
	Tot GHG emissions (Mt CO_2_-eq.)	26.02	24.52	25.22
TOT GHG emissions (Mt CO_2_-eq.)		195.23	181.58	167.04

## Discussion and Conclusions

This manuscript presents an extension to the work of ‘*Greenhouse gases and natural capital implications of plastics and bioplastics*’ of the 2020 ETC/WMGE work programme by the same authors. The majority of the work, the 2018 GHG emission scenario model and inventories for GHG factors, have been produced within the task during the project. The extension is the future 2025 model and the estimation of the GHG emission reduction potential of the European Union’s circularity related targets. The overall goal of this study was to map the current plastic flows and related GHG emissions for the total plastics value chain in the EU currently (2018) and in the near future (2025) by which several targets should be met. The aim of the work was to improve the understanding of the correlation between different phases and forms of the plastics value chain and climate impacts, and what kind of a role circular plastics economy could play in addressing the impact on climate change and mitigation of those impacts in Europe.

Attempts to map material flows and related GHG emissions for the total plastics value chain globally and EU-wise are scarce. Plastics are made from different raw materials via different methods, and furthermore, they are used in a wide range of applications that have different fates once they are discarded from use. As the whole value chain including the wide range of varying plastic applications, mapping is a complex effort, and it is complicated to track the fate of carbon-containing resources through feedstock and polymer production to a variety of plastic products with different lifetimes and end-of-life options.

In this study, GHG emissions related to the European plastics value chain in 2018 and future 2025 were estimated using a bottom-up approach to understand the current GHG emissions and impact, and possible future GHG emissions and impact to climate change. As data for the models are collected from several sources as well as several assumptions are made, the data quality and model uncertainties were estimated. The models present a cautious estimation of what the GHG emissions were in 2018 and how by 2025 they could change if circular actions are taken. To analyse the uncertainty of the 2025 model, two alternative scenarios (pessimistic and optimistic) were created to estimate the overall GHG emissions if targets would not be met or if they would be exceeded.

### Comparison of Models

The models ideally are able to provide insights to inform future discussions on the potential and limitations of circular plastics and the corresponding impacts on climate. The current model based on figures for 2018 estimates the GHG emissions in a mostly linear plastics value chain. A future scenario was further built to estimate the overall GHG emissions in 2025, when the plastics value chain should have changed according to the European Plastics Strategy and European legislation.

The bottom-up analysis started from plastic production, consumption and waste management data, with GHG factors then attributed to the flows in each lifecycle step, which then allow the calculation of the emissions throughout the value chain. There is considerable variability among specific plastics in GHG emissions during their lifetimes, depending on the polymer type’s production and conversion techniques. Currently, polymers are mainly produced from fossil sources like crude oil. However, recycled plastics and renewable raw materials as biomass will become ever more important feedstock streams for the circular plastics economy.

The total GHG emissions caused by the plastics value chain, for the plastics volume converted in the EU in 2018, are estimated at 208 Mt of CO_2_-eq. The majority, 63%, of the GHG emissions in the EU plastics value chain are caused by its production. Converting these polymers into products accounts for 22%, and plastic waste treatment at end-of-life adds another 15%, mainly due to incineration.

As the total GHG emissions in the EU in 2018 were 3893 Mt CO_2_-eq. [46], the EU plastics value chain contributes to approximately 5.5% [8]. It has been estimated by Zheng and Suh (2019) that the global plastic production was 380 Mt in 2015 producing emissions of 1.8 Gt of CO2-eq. [37]. For comparison, the total plastics production in Europe in 2018 was 61.2 Mt, which is around 16% of the total global production. If we apply this share (16%) to the Zheng and Suh (2019) figure of 1.8 Gt CO_2_-eq., then the GHG emissions for the EU would be estimated at 285 Mt CO_2_-eq.

**Fig. 6:**
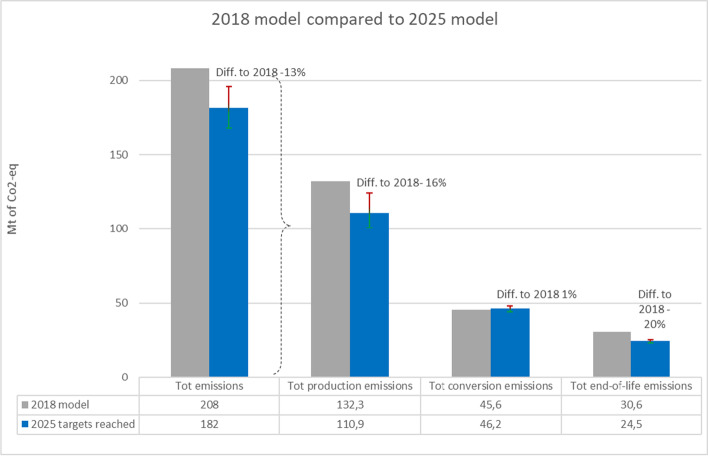
Comparison of the GHG emissions along the plastics value chain between the 2018 and 2025 models

As the current plastic value chain’s (2018) GHG emissions are 208 Mt of CO_2_-eq., the saving potential estimation for the EU’s plastics value chain is 26 Mt of CO_2_-eq. or approximately 13% with the current actions to support plastic waste recycling, use of plastic recyclates, and decreasing the amount of landfilling of plastic waste (2025 on target). With the positive and negative scenarios for 2025, the savings could be − 20% and − 6% respectively when compared to 2018. A comparison of the models for 2018 and 2025 is presented in Fig. 6.

The plastics value chain has thus untapped potential to reduce related GHG emissions due the current overall low recycling rate and high incineration rate. Demand for recycled plastics today account for only 6% of the plastic demand in Europe, so the recycled plastics market is rather underdeveloped and there is a need to produce high-quality recyclates to supply to the markets. Overall for both models, the energy-related emissions from heat, steam and electricity production are a major contributor to CO_2_ emissions in the plastics value chains. As a large share of the emissions originate from production of polymers, the biggest potential for GHG emission reduction lies there. This can be efficiently targeted by recycling and utilising recyclates in components and products. The 2025 model supports the hypothesis that high-quality recycling of plastics is beneficial to climate impact in two ways:

- It avoids CO2 emissions during incineration of the plastic waste; and
- Recyclates avoid the use of primary raw materials and therefore avoid the corresponding GHG emissions.

Overall, when 1 kg virgin fossil-based plastic product enters the markets, it has already caused at least 2.9 kg of GHG emissions and it will cause a further 2.7 kg of GHG emissions when it is discarded and if it is incinerated [8]. If the recycling process leads to a recyclate that can be used for the production of new products, this recyclate displaces virgin plastic on the market, reducing raw material extraction and production of virgin plastics, which are mainly fossil-based, and therefore leads to reduced GHG emissions. Based on the estimates the avoided emissions from virgin polymer production from fossil resources could be 1.91–5.70 kg CO_2_-eq./kg polymer [8]. This is in line with other sources in the literature, which claim that recycling rather than incinerating plastic could reduce emissions by 1.1–3.0 tonnes CO_2_-eq./tonne plastic compared to plastics from virgin fossil feedstock [18]. In the future 2025 model, the avoided emissions were taken into account by excluding production-related emissions for 10 Mt of recycled polymers with a GHG factor of 2.25 kg CO_2_-eq./kg polymer.

### Data Quality and Model Uncertainty

There are uncertainties in the data used for this study (Table 11). Volume data and emission data were extracted from multiple sources, which do not provide a clear indication of accuracy. Where possible, data representative for the EU were selected. Assumptions were however made to simplify the processes involved in plastic lifecycles and value chains. Yet, no better data sources were found.

**Table 11:** Table quality—table overview

Data	Figure	Source	Underlying assumptions	Data quality
**2018**				
**Volumes (Mt)**				
Crude oil production and refinery	51.1	Plastics Europe, 2019. Plastics, The Facts (EU28 + NO/CH total converters demand* for 2018). Includes thermoplastics, polyurethanes, thermosets, elastomers, adhesives, coatings and sealants, and PP-fibres. Not included are: PET-fibres, PA-fibres and polyacryl-fibres	EU converters demand in EU as a measure for crude oil demand allocated to EU plastic chain	No quantitative indication of data accuracy. Data was collected by PlasticsEurope (the Association of Plastics Manufacturers in Europe) and EPRO (the European Association of Plastics Recycling and Recovery Organisations). PlasticsEurope’s Market Research and Statistics Group (PEMRG) provided input on the production and the demand of plastic raw materials. Conversio Market & Strategy GmbH helped assess waste collection and recovery data. Official statistics from European or national authorities and waste management organisations have been used for recovery and trade data, where available. Research or expertise from consultants completed gaps
PP (production and conversion)	9.9	Plastics Europe, 2019. Plastics, The Facts (EU28 + NO/CH total converters demand* for 2018). Includes thermoplastics, polyurethanes, thermosets, elastomers, adhesives, coatings and sealants, and PP-fibres. Not included are: PET-fibres, PA-fibres and polyacryl-fibres	EU converters demand in EU as a measure for resin production demand for EU consumption	
PE-LD (production and conversion)	9			
PE-HD (production and conversion)	6.2			
PVC (production and conversion)	5.1			
PUR (production and conversion)	4			
PET (production and conversion)	3.9			
PS/EPS (production and conversion)	3.3			
Others (production and conversion)	9.7			
Recycling	9.4	Plastics Europe, 2019. Plastics, The Facts (Waste Collection and treatment figures for 2018)	Part of the plastics stay in-use and are stocked, or littered—no data on these volumes	
Incineration (energy recovery)	15.8			
Landfilling	7.2			
Carbon intensity factors (kg CO_2_-eq./kg plastic)				
Resource extraction	0.228	Eco-profiles and environmental product declarations of the European Plastics Manufacturers. Polypropylene (PP). PlasticsEurope. April 2014. December 2016: update water balance. (www.plasticseurope.org/en/resources/eco-profiles)	Average carbon intensity for the EU cracker capacity mix, as estimated in kg CO2 per kg of crude oil, taken as a proxy for fossil resources allocated to the production of plastics	No quantitative indication of data accuracy. Environmental Product Declaration reviewed by DEKRA Consulting GmbH and approved according to the Product Category Rules PCR version 2.0 (2010–06) and ISO 14025:2006
Refinery	0.34	Eco-profiles of the European Plastics Industry. Naphtha. A report by I Boustead for Plastics Europe. March 2005. (www.plasticseurope.org/en/resources/eco-profiles)	Refinery step, estimated in kg CO2 per kg of naphtha, taken as a proxy for fossil resources allocated to the production of plastics	No quantitative indication of data accuracy
PP production	1.91	Eco-invent v.3.6. record: Polypropylene, granulate production (Europe)	Values include emissions related to European electricity for cracking, polymerisation processes which take place in the EU, and world energy mixes for upstream processes such as crude oil refining	No quantitative indication of data accuracy. Eco-invent database quality requirements apply
LD PE production	1.98	Eco-invent v.3.6. Polyethylene, low density, granulate production(Europe)		
HD PE production	1.93	Eco-invent v.3.6. record: Polyethylene, high density, granulate (RER) production (Europe)		
PVC production	2.51	Eco-invent v.3.6. record: Polyvinylchloride bulk polymerised production (Europe)		
PUR production	5.7	Eco-invent v.3.6. record: Polyurethane, rigid foam production (Europe)		
PET production	2.94	Eco-invent v.3.6. record: Polyethylene terephthalate, granulate, amorphous production (Europe)		
PS production	3.68	Eco-invent v.3.6. record: Polystyrene, general purpose production (Europe)		
Other	2.51	Own calculation	Median value of PP, LD PE, HD PE, PVC, PUR, PET, PS production records (see higher)	No quantitative indication of data accuracy
PP conversion	0.94	Eco-invent v.3.6. (Conversion technology data records: Injection mould processing (Europe); blow mould processing (Europe); Stretch blow moulding (Europe); calendering, rigid sheets (Europe); extrusion of plastic film (Europe); extrusion of plastic pipes (Europe); polymer foaming processing (RER); thermoforming with calendering (Europe))	74% injection moulding; 24% blow moulding; 2% extrusion (pipes) (Split based on Zheng and Suh, 2019, Keoleian, 2012)	No quantitative indication of data accuracy. Eco-invent database quality requirements apply
LD PE conversion	1.13		67% injection moulding; 24% blow moulding; 9% extrusion (pipes) (split based on Zheng and Suh, 2019, Keoleian, 2012)	
HD PE conversion	1.13		67% injection moulding; 24% blow moulding; 9% extrusion (pipes) (split based on Zheng and Suh, 2019, Keoleian, 2012)	
PVC conversion	0.51		51% extrusion (pipes); 18% calendering (sheets); 29% injection moulding; 2% blow moulding (split based on Zheng and Suh, 2019, Keoleian, 2012)	
PUR conversion	0.51		100% polymer foaming (split based on Zheng and Suh, 2019, Keoleian, 2012)	
PET conversion	0.94		50% injection moulding; 50% blow moulding (split based on Zheng and Suh, 2019, Keoleian, 2012)	
PS conversion	0.51		100% polymer foaming (split based on Zheng and Suh, 2019, Keoleian, 2012)	
Other	0.94	Own calculation	Median value of PP, LD PE, HD PE, PVC, PUR, PET, PS conversion values as described higher	No quantitative indication of data accuracy
Recycling	0.32	Plastics Recyclers Europe (2015)	Average GHG emission value for mechanical recycling, based on value for PET (0.510 kg CO^3^ eq./kg waste) and other plastics (0.348 kg CO_2_-eq./kg waste), and adding GHG emissions for collection (0.017 kg CO_2_-eq./kg waste), sorting (0.027 kg CO_2_-eq./kg waste) and transport to recyclers (0.022 kg CO_2_-eq./kg waste). Interval: 0.269–0.373 kg CO_2_-eq./kg waste	No quantitative indication on data accuracy
Incineration	2.71	Plastics Recyclers Europe (2015) and ETC WMGE. 2021. ‘Plastic in Textiles: Potentials for Circularity and Reduced Environmental and Climate Impacts.’ https://www.eionet.europa.eu/etcs/etc-wmge/products/plastic-in-textiles-potentials-for-circularity-and-reduced-environmental-and-climate-impacts	Own calculation. As the energy content of plastic waste is generally recovered during incineration (as electricity, heat or both), the incineration process is credited for the benefits from avoided production of conventional energy (electricity and heat) replaced by energy recovered from plastic waste incineration. These avoided emissions are calculated based on EU average energy efficiencies for waste incineration of 13.7% for electricity production and 31.8% for heat recovery, and on EU average data for conventional electricity and heat production (as in the EU greenhouse gas emission inventory and the Ecoinvent database). (Nessi 2020b) (Nessi 2020a) These credited or avoided emissions apply both to plastic waste sent directly to incineration and to sorting and reprocessing residues of plastic waste sent to recycling	No quantitative indication on data accuracy
Avoided emissions due to energy recovery at incineration	0.98			
Landfilling	0.03		The available literature on greenhouse gas impacts of landfilling plastic waste gives a range of 0.004–0.010 tonnes of CO2-eq./tonne of plastic waste (Deloitte and Plastics Recyclers Europe, 2015). Assuming a value of 0.01 tonnes of CO2-eq./tonne of plastic waste, and adding 0.017 tonnes of CO2-eq./tonne for the collection of the waste (see above), a value of 0.03 tonnes of CO2-eq./tonne for the landfilling plastic waste can be assumed	No quantitative indication on data accuracy
Additional data for 2025 model				
Carbon intensity factors (kg CO_2_-eq./kg plastic)				
PLA production	0.12	The bio-based polymer average for GHG emissions for cradle to gate has been calculated based on a literature review and case studies. Spearling et al. (2018) reviewed 29 lifecycle analyses of bio–based plastics in which biogenic carbon was included. In all of these studies, GWP was in the range of − 0.3–11.9 kg CO2-eq./kg for bio-based plastics. *Sources used:* (Blanco, Ingrao, and Siracusa 2020), (Brizga, Hubacek, and Feng 2020), (Dilkes-Hoffman et al. 2019), (Pendrill et al. 2019), (Spierling et al. 2018), (CE Delft 2017), (Vink and Davies 2015), (L. Shen, Worrell, and Patel 2012) and (Kim and Dale 2008) A literature review has been made to match the fossil plastics with bio-based plastic alternatives. *Sources used for matching the bio-based alternatives to fossil-based counterparts:* Blanco et al. (2020), Brizga et al. (2020), EASAC (2020), Dilkes-Hoffman et al. (2019), Morão and de Bie (2019), Lewandowski (2018) and Spierling et al. (2018)	A big uncertainty in this estimate lies in the fact that the range of greenhouse gas emissions for bio-based plastic value chains was collected from different case studies that are challenging to compare due to differences in the methodologies used and that the polymer-based averages used may have rather high deviance. Furthermore, the generalisation of fossil- versus bio-based replaceability does not fully represent reality. The complexity of bio-based value chains needs to be taken into consideration when evaluating the simplified estimation	No quantitative indication on data accuracy
PBAT production	3.11			
PE production	0.9			
PE-HD conversion	1.16			
PLA conversion	0.12			
PBAT conversion	0.22			
EoL composting	1.49	The end-of-life-management GHG emissions related to each step were gathered so that landfilling, incineration and recycling have the same factor as the fossil-based section of this report, and the composting was reviewed from literature A study by Hermann et al. (2011) calculated the net GWP of incineration of some biodegradable polymers (PLA, starch/PCL, starch, PHBV, PBAT) with an average of 1.97 kg CO2-eq./kg of material without energy recovery and 1.25 kg CO2-eq./kg of material with energy recovery. The incineration of bio-based plastics has similar emissions to fossil-based plastics: due to the limited amount of information on incineration of bio-based plastics, 2.71 kg CO2-eq./kg of material was used in this study for the calculation of fossil-based and bio-based plastics incineration for direct and indirect emissions, and − 0.976 kg CO2-eq./kg for avoided emissions because of energy recuperation Based on one study case, the greenhouse gas emissions from composting biodegradable polymers (PLA, starch, starch/PCL, PHBV, PBAT) were on average 1.62 kg CO2-eq./kg of material. (Hermann et al. 2011). Avoided emissions for composting are estimated at − 0.133 kg CO2/kg waste (OVAM 2020)	To approximately quantify the greenhouse gas emissions from the existing and potential bio-based plastics value chains, the different stages were reviewed separately. The emissions from the conventional plastics’ value chain have been applied to those parts of the bio-based value chain that are similar—conversion, mechanical recycling, incineration and landfilling. An average of fossil-based plastics’ greenhouse gas emissions from conversion, recycling, incineration and landfilling has been used for bio-based plastics calculations as well The data used is based on a few data sets from mentioned study and also the avoided emissions for composting is based on one source. Uncertainty in this estimate lies in the fact that there are not many data points and that the data has been collected from different studies and reports	No quantitative indication on data accuracy

The lack of insight in quantitative uncertainty analysis in Life Cycle Assessment results (which were also used as a basis in this report) is a problem which is acknowledged in literature. Lloyd and Ries (2007) distinguish three main categories of uncertainty in LCA: model, scenario and parameter uncertainty. Parameter uncertainty (also called stochastic or data uncertainty) is the uncertainty in observed or measured values arising from inherent variability in the sampled population, as well as uncertainty related to data quality. Scenario uncertainty refers to uncertainty due to choices made in establishing scenarios. Model uncertainty comes from the structure of and the mathematical relationships defining the LCA models (including models for deriving emissions and characterisation factors used in impact assessment models).

Lloyd and Ries (2007) further give an overview of suggested approaches for estimating unknown uncertainty in LCA input parameters. These show standard deviations which are typically in the range of 20 to 30% for lower quality data, for example regarding emissions. Different approaches lead to a wide range of uncertainty estimates and are usually selected through the experience and best judgement of the individuals involved in the different studies. Transforming data quality indicators directly into probability distributions may be inaccurate. Unless distribution forms and parameters are defined, information on data quality does not provide a basis for quantitative accuracy assessments [47].

From basic error propagation rules, we know that if x and y have independent random errors $\partial x$ and $\partial y$ then the absolute error in (1):

1 $$z=x+y \mathrm{is }\partial z=\sqrt{\left(\partial {x}^{2}+\partial {y}^{2}\right)}$$

Similarly, the relative error in (2):

2 $$z=x\times y \mathrm{is }\frac{\partial z}{z}=\sqrt{{\left(\frac{\partial x}{x}\right)}^{2}+{\left(\frac{\partial y}{x}\right)}^{2}}$$

Starting from a relative random error of 10% in the plastic conversion volume data and a random error of 10% in the CO_2_ intensity factors per polymer would thus result in an estimated GHG emissions with a standard deviation of 14%. If initial errors on both factors would be 20%, the resulting product of the multiplication would have a relative error of 28%. Yet, since no better data sources were found, and accuracy of the volumetric data and Life Cycle data is not known, calculations were made based on the available estimates, and without further uncertainty analysis. Resulting figures should thus be considered as indicative.

A review of LCA studies done by Bamber et al. (2019) showed that the vast majority of LCA studies (79%) did not perform any kind of uncertainty analysis. Among the reviewed studies, Monte Carlo is the most common method of uncertainty analysis in attributional LCA, accounting for 60% of LCA studies that reported uncertainty analysis. Monte Carlo analysis is typically used for parameter uncertainty. However, other important sources of uncertainty are often overlooked. This is an important issue since including some, but not all sources of uncertainty, can lead to incorrect and misleading results [48, 49]. Weidema et al. (2013) come to a similar conclusion as they discuss data quality guidelines for the Ecoinvent 3 database (which was used also in our study) [50].

Some guidelines are available to assess default basic uncertainty applied to intermediate and elementary exchanges documented in the Ecoinvent database: for pollutants emitted to the air, like CO2. In addition to this basic uncertainty, an additional uncertainty from data quality indicators is added to the lognormal distribution. Model uncertainties and human errors are however not accounted for. Also, no uncertainty values are provided for the impact assessment results from the database. Current impact assessment methods do not provide information on uncertainty. Moreover, the contribution of the uncertainty in damage factors to the overall impact assessment results is judged to be at least as important as the uncertainty in the LCI results. Showing uncertainty values on the level of the LCIA results without considering the LCIA uncertainties would be misleading.

### Research Gaps

From both of the models, it is evident that the amount of annual stock up, losses, littering, etc., is rather high, as there is a disequilibrium between annual production/conversion and end-of-life volumes (which is not solely explained by trade balances). Literature does not offer a clear answer on fate of plastic products and waste. It is however clear that different lifetimes of different plastic products affect the amount of annual storage, for example stock is generated in buildings and automotive. Plastic waste can end up in mixed waste streams where it does not get accounted for. The models are based on registered data, so reporting most likely also play a role in explaining the amounts stored annually. The fate of these material stocks (in the urban mine, in landfills, in litter…) on the long term is unclear, and their emissions are unknown and therefore not accounted for. This unknown data gap presents challenges in mapping and calculations and would require more research.

Reliable and unambiguous data about waste volumes and materials management are important for the monitoring of greenhouse gas emissions at a value chain level. Methods for calculating, verifying and reporting European data on waste were recently changed by the Commission’s decision 2019/1004 and will be applicable to data from the year 2020 onwards [52]. Up until now, member states have calculated and reported the quantity of plastic waste for example at different points in the collection, sorting and recycling process, which has led to higher reported recycling rates than what has actually been recycled. The change in the calculation method does not affect the 2025 model produced per say as it is estimated that the total 10 Mt of recyclates would enter the markets, but it is notable that to reach the 2025 recycling targets will most likely require further efforts from the value chain than initially thought.

Data on uses of plastics and the related environmental impacts are disparate, for which they were excluded in this study. Yet, an attempt to include also this in future research would be important. For example, plastic packaging’s role in avoiding food losses and waste is recognised—food losses and waste account for 4.4 Gt of CO_2_-eq., which is approximately 6% of global GHG emissions [51]. Lower weight cars with a higher use of plastics can result in lower consumption of petrol, or buildings insulated with plastic-based materials consume less energy during their lifetime. How to deal with these cross-sectoral effects in emission-accounting is still subject of discussion. Further research is needed to be able to take into account the use phase environmental impacts.

Furthermore, an important aspect that is not included in the calculations is how reuse models and decrease of single-use products due to the SUP directive will affect the overall GHG emissions in the future. With reuse models, recycling and manufacturing steps are avoided, so GHG emissions can be substantially reduced. Activities to eliminate the use of unnecessary plastics or plastics that are challenging to recycle through redesign will decrease the amount of plastic demand and waste. SYSTEMIQ in their recent report (2022) have estimated that there could be almost a 5 Mt reduction of plastic waste by 2030 through these activities [52].

Also, innovative recycling processes, such as chemical and thermochemical recycling, are in development and may have the potential of complementing mechanical recycling and substituting incineration of plastic waste. This could further lead to reduction in the future GHG emissions. SYSTEMIQ in their recent report (2022) have estimated that chemical recycling could scale to 3 Mt by 2030. As (thermo)chemical recycling is emerging to the markets and volumes are most likely to scale in the future, it is important to research the sustainability and create open access data on the GHG emissions of these additional and alternative end-of-life-treatment options.

As of today, the current share of bio-based polymers in total plastics production at 1% is very small. The potential of bio-based plastics to reduce plastics’ carbon footprint lies mainly in sourcing because of the sequestration of CO_2_ during the production of biological raw materials [8]. It is however important to consider the circularity and the suitability of bio-based plastics in the current recycling and industrial composting infrastructure to avoid recyclable bio-based ending up in incineration. If incinerated, the sequestered carbon is just released again and the net result of sourcing and end-of-life is zero. Production, conversion, distribution and use still have a certain carbon footprint, although lower when compared to fossil-based plastics.

Overall, the value chains for specific polymers of both virgin and recycled fossil-based plastics as well as bio-based plastics are very complex, and the related GHG emissions vary a lot depending on the raw materials, the production and utilisation of side and waste streams, and the different end-of-life options. The reduction potential of 26 Mt or 13% of the future model is rather moderate. On the other hand, taking into account that the savings for the plastics value chain are annual, not a onetime thing, the cumulative reduction can be considered to be more substantial. It is however clear that the plastics value chains contribute significantly to climate change now and in the future.

Currently, the plastics value chain contributes to about 5.5% of EU’s annual GHG emissions. In 2025, the EU is targeting a 15% reduction in its emissions, which would mean that the overall GHG emissions should be approximately 3309 Mt CO_2_-eq. In this case, the future model, 182 Mt CO_2_-eq, would still however account for 5.5% of EU’s annual GHG emissions. Recycling and using recyclates to replace virgin fossil feedstock are key aspects both in the circular economy of plastics as well as in reducing the GHG emissions of the plastics value chain, whilst further reduction potential should be sought from decreasing the amount of plastic waste generated, especially by limiting the use of single-use products, and by implementing reuse business models.

Building models and scenarios is challenging for the very complex plastics value chains, which is further challenged by lack of consistent and comprehensive data. There are significant data gaps, which was evident in the model and scenario exercises carried out in this study. To be able to in a better way estimate and monitor the effects of circularity as well as impacts to environment and GHG emissions, it would be important to improve the data collected on plastics in Europe and develop more advanced methods for quantifying the GHG emissions for circular plastics.

## Data Availability

The data used are shared in the tables presented. Further data is available from the references stated. The data gathered and analysed for the studies are included in this published article. The raw data can be reasonably requested from the corresponding author.

## Abbreviations

(E)PS: (Expanded) polystyrene
Bio-PE: Bio-based polyethylene
CAGR: Compound annual growth rate
CE: Circular economy
CO2: Carbon dioxide
CO2-eq: Carbon dioxide equivalent
EEA: European Environmental Agency
ETC/WMGE: European Topic Centre Waste and Materials in Green Economy
GHG: Greenhouse gas
Gt: Gigatonnes
GWP: Global warming potential
HD-PE: High density polyethylene
IPCC: International Panel on Climate Change
IR: Infrared radiation
kg: Kilogramme
kt: Kilotonne
LD-PE: Low density polyethylene
LHV: Lower heating value
MJ: Megajoule
Mt: Million tonnes
PA: Polyamides
PBAT: Polybutylene adipate terephtalate
PE: Polyethylene
PET: Polyethylene terephthalate
PLA: Polylactic acid
PP: Polypropylene
PUR: Polyurethane
PVC: Polyvinylchloride
VITO: Flemish Institute for Technological Research
VTT: Technical Research Centre of Finland Ltd
EU: European Union
